# A Case Report of a Boy With Clinically Mild Encephalopathy and a Reversible Splenial Lesion Associated With Severe Acute Respiratory Syndrome-Coronavirus 2 Infection

**DOI:** 10.7759/cureus.80241

**Published:** 2025-03-08

**Authors:** Yuko Moriuchi, Tatsuo Fuchigami, Ichiro Morioka

**Affiliations:** 1 Department of Pediatrics, Itabashi Medical System (IMS) Fujimi General Hospital, Fujimi, JPN; 2 Department of Pediatrics and Child Health, Nihon University School of Medicine, Tokyo, JPN

**Keywords:** child, covid-19, diffusion-weighted imaging, mild encephalitis/encephalopathy with a reversible splenial lesion (mers), sars-cov-2

## Abstract

Severe acute respiratory syndrome coronavirus 2 (SARS-CoV-2) infection is associated with a high frequency of central nervous system abnormalities, particularly acute encephalopathy, in children. We report the case of a nine-year-old boy with SARS-CoV-2-associated clinically mild encephalopathy with a reversible splenial lesion. The patient was admitted to our hospital with fever, vomiting, and poor speech. The patient tested positive for SARS-CoV-2 by polymerase chain reaction of a nasal swab sample. The cerebrospinal fluid cell count was normal. The patient had a low serum sodium level upon admission. Computed tomography of the brain revealed mild cerebral edema. Diffusion-weighted magnetic resonance imaging (MRI) revealed transient abnormally high signal intensity in the splenium of the corpus callosum. Electroencephalography revealed generalized high-voltage slow waves. The patient was clinically diagnosed with mild encephalopathy and a reversible splenial lesion associated with SARS-CoV-2 infection. The patient was discharged without any neurological sequelae. In conclusion, it is useful to perform MRI evaluations in children with SARS-CoV-2 infection and impaired consciousness, poor speech, and behavior, considering the possibility that they may be in the early stages of encephalopathy.

## Introduction

Coronavirus disease 2019 (COVID-19), caused by severe acute respiratory syndrome coronavirus 2 (SARS-CoV-2), primarily produces respiratory symptoms. However, since the sixth wave, when the Omicron strain became the mainstream of the epidemic, higher frequencies of central nervous system abnormalities, especially acute encephalopathy due to pediatric COVID-19, have been reported [1,2]. Tada et al. [3] identified clinically mild encephalitis/encephalopathy with a reversible splenial lesion (MERS). It was characterized by transient splenial lesions with high signal intensity on diffusion-weighted magnetic resonance imaging (MRI), a mild clinical course, and a good outcome as a new type of acute encephalopathy [3]. A reversible splenial lesion in the corpus callosum with homogenous reduced diffusion may be detected in various conditions such as infection, withdrawal of antiepileptic drugs, altitude sickness, Kawasaki disease, electrolyte abnormalities (especially hyponatremia), hypoglycemia, and X-linked Charcot-Marie-Tooth disease. In particular, encephalitis and encephalopathy with mild neurological symptoms and good prognosis have been reported as MERS [4].

The most common MERS pathogen is the influenza virus (22%), followed by rotavirus (9%) and human herpesviruses 6 and 7 (5%) [5]. However, few reported cases of MERS have been associated with SARS-CoV-2 infection in children [6-9].

Here, we report the case of a nine-year-old boy with MERS associated with SARS-CoV-2 infection.

## Case presentation

A previously healthy nine-year-old boy presented to another clinic with a fever, followed by vomiting and appetite loss, and the patient was then admitted to our hospital on the same day. He had no history of seizures and his development was normal.

On the day of admission, the patient had a fever of 39.1 ℃. His blood pressure was 103/57 mmHg, his pulse rate was 123 beats/min, and his oxygen saturation was 99% from room air. His general health status was somewhat poor. The patient’s speech was affected, and his consciousness was 13 points on the Glasgow Coma Scale (GCS) of E4 V3 M6. Neurological examination revealed no abnormalities, except for his poor speech. No neck stiffness was observed. The chest and abdomen exhibited no abnormalities. The patient had no cough, respiratory problems, or abnormal findings on the chest radiograph. The capillary refilling time was elevated (3 s).

Initial laboratory blood findings revealed normal leukocyte and platelet counts and elevated C-reactive protein at 2.08 mg/dL. The serum electrolytes were low sodium at 133 mEq/L and low chloride at 96 mEq/L. Blood urea nitrogen was elevated at 20.8 mg/dL, aspartate aminotransferase at 51 IU/L, and lactate dehydrogenase at 260 IU/L (Table 1).

**Table 1:** Investigation results

Laboratory blood findings	Our patient’s results	Reference range
Leukocyte cell (/µL)	6,300	3,300–8,600
Platelet (/µL)	199,000	158,000–348,000
C-reactive protein (mg/dL)	2.08	0.01–0.14
Sodium (mEq/L)	133	136–147
Potassium (mEq/L)	4.3	3.5–5.0
Chloride (mEq/L)	96	98–108
Urea nitrogen (mg/dL)	20.8	8.0–23.0
Creatinine (mg/dL)	0.71	0.61–1.08
Aspartate aminotransferase (IU/L)	51	8–40
Alanine aminotransferase (IU/L)	31	5–45
Lactate dehydrogenase (IU/L)	260	124–222
Creatinine kinase (IU/L)	91	55–250
Glucose (mg/dL)	71	70–109
Ammonia (µg/dL)	25	55–250
Cerebral spinal fluid results		
Leukocyte cell (/µL)	5	0–5
Protein (mg/dL)	32.1	10–40
Glucose (mg/dL)	53	50–75

The patient had no history of SARS-CoV-2 infection and had not been vaccinated against SARS-CoV-2. Approximately two weeks prior, his mother had tested positive for SARS-CoV-2 by polymerase chain reaction (PCR) of a nasal swab sample.

**Figure 1:**
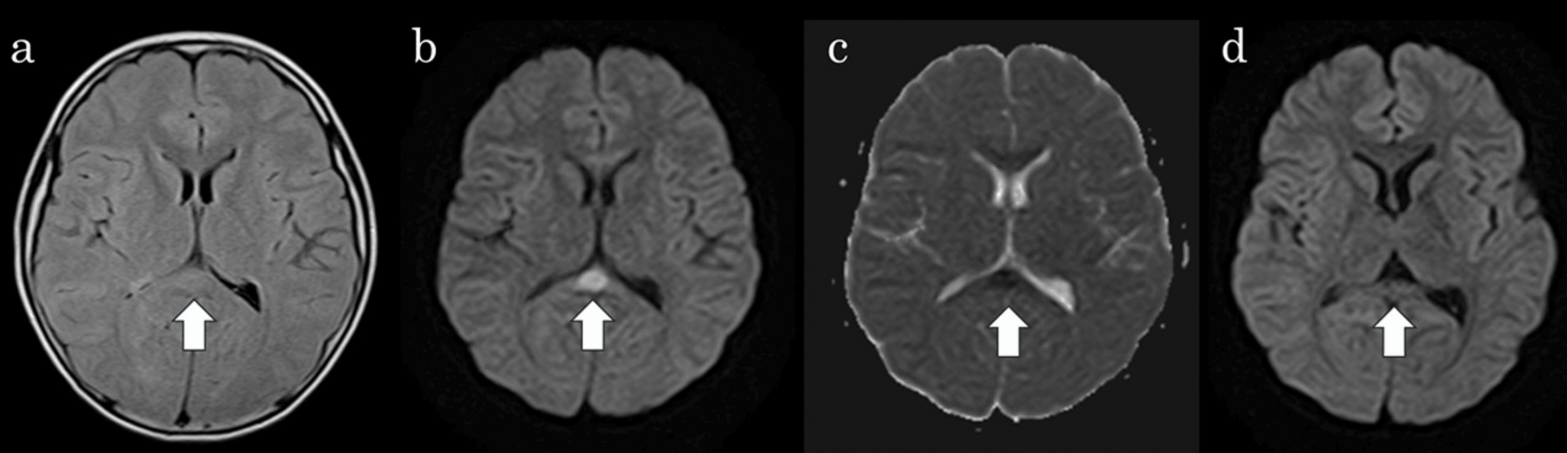
Brain MRI Initial MRI revealed abnormal signals in the splenium of the corpus callosum (arrow), which were of somewhat hyperintensity on fluid-attenuated inversion recovery imaging (a), hyperintensity on diffusion-weighted imaging (DWI) (b), and hypointensity on apparent diffusion coefficient (ADC) mapping (c). Follow-up MRI showing complete improvement of the SCC on DWI (arrow) (d). SCC: Splenium of the corpus callosum

The patient tested positive for SARS-CoV-2 by PCR of a nasal swab sample. Cerebral spinal fluid examination showed no abnormality. Brain computed tomography (CT) revealed mild edema. A brain MRI was performed on the second day of admission because of persistent poor speech. Abnormal signals were observed, however, in the splenium of the corpus callosum (SCC), which revealed a somewhat high intensity on fluid-attenuated inversion recovery imaging (Figure 1a), hyperintensity on diffusion-weighted imaging (DWI) (Figure 1b), and apparent diffusion coefficient (ADC) mapping showed decreased ADC values at the abnormality (17.2 mm^2^/s) (Figure 1c).

**Figure 2:**
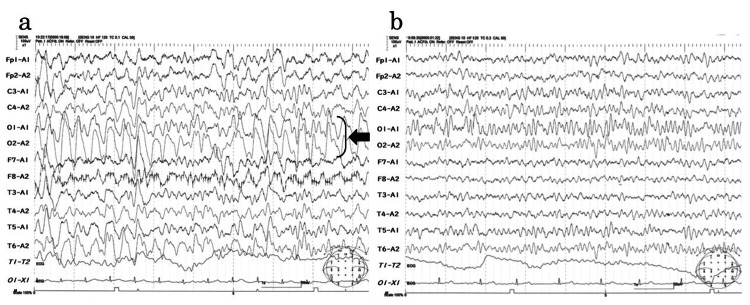
EEG a. Awake patient (second day of admission) exhibited diffused high-voltage slow waves of approximately 2–3 Hz in the particularly marked occipital area (arrow). Calibration: 100 µV, 1 s b. The patient was awake (ninth day of admission) and provided almost normal findings, with no slow waves. The background rhythm was 9–10 Hz, there was no laterality, and the normal spindle and hump were shown. Calibration: 100 µV, 1 s

Magnetic resonance angiography did not reveal any cerebral vascular stenosis or aneurysms. Electroencephalography (EEG) on the second day of admission revealed diffuse high-voltage slow waves during awake recordings (Figure 2a).

These findings strongly suggest that acute encephalopathy is associated with SARS-CoV-2 infection. The patient was administered intravenous osmotic diuretic and edaravone. He could state his name and age on the third day of admission, speak spontaneously on the fourth day, and respond to questions on the fifth; therefore, methylprednisolone pulse therapy was not started. On the eighth day after admission, follow-up MRI revealed complete improvement in the SCC signal changes (Figure 1d). The EEG on the same day was almost normal. No slow waves or seizures were observed (Figure 2b). The final diagnosis was MERS associated with SARS-CoV-2 infection. On the 11th day of admission, the patient was discharged without any neurological sequelae. The patient’s development and intelligence have remained unchanged since discharge.

## Discussion

During national surveillance from 2014 to 2017 (during the previous SARS-CoV-2 infection outbreak) in Japan, 1115 acute encephalopathies associated with infectious diseases occurred in Japanese children and were most common in infants aged 0-3 [10]. These have been classified into several clinic-radiological syndromes, such as acute encephalopathy with biphasic seizures and late reduced diffusion, which is the most common subtype (34.0%), followed by clinically mild encephalitis/encephalopathy with a reversible splenial lesion (MERS) (19.3%) and acute necrotizing encephalopathy (2.8%) [10]. However, in a nationwide epidemiological study during the SARS-CoV-2 infection outbreak in Japan, MERS accounted for 8.7% of the encephalopathy syndromes due to SARS-CoV-2, which is lower than the 19.3% of non-SARS-CoV-2-associated encephalopathies [11]. Patients with MERS typically exhibit symptoms associated with the central nervous system, including delirious behavior, consciousness disturbances, and seizures, with complete recovery within a month [5]. It has been speculated that the low number of SARS-CoV-2-associated MERS cases may be due to the difficulty in obtaining MRI scans in COVID-19 patients due to isolation after hospitalization, which may result in transient lesions in the splenium being missed, which are essential for the diagnosis of MERS [3,11]. In addition, the mean age of patients with MERS in conventional viral encephalopathy is 5.6 years [10], whereas the patients in this study were relatively older at nine years of age. Table 2 summarizes the 10 cases of MERS associated with SARS-CoV-2 infection in children that have been reported [6-9].

**Table 2:** Summary of MERS associated with SARS-CoV-2 infection in previously published reports F: female; M: male; MIS-C: multisystem inflammatory syndrome in children; MRI: magnetic resonance imaging; NR: not reported; PR: present report

Patient number	Reference	Age (years)	Sex	Neurological symptoms	Serum sodium (mEq/l)	Day of the first MRI (since the first neurological symptoms)	Day of follow-up MRI (since the first neurological symptoms)	Diagnosis	Outcome
1	Abdel-Mannan et al. [6]	15	F	Confused, disoriented, headache, weakness	Normal	5 days	NR	MIS-C	Recovering
2	Abdel-Mannan et al. [6]	15	F	Confused, dysarthria, dysphagia	Normal	21 days	NR	MIS-C	Day 32: still inpatient; encephalopathy resolved, wheelchair bound
3	Abdel-Mannan et al. [6]	9	M	Confused, ataxia, dysarthria, headache	129	1 day	6 days (with minimal signal changes remaining on T2)	MIS-C	Recovering
4	Varol et al. [7]	15	M	Headache, dizziness, blurred vision, agitation, vomiting	128	5 days	15days (disappeared)	MIS-C	Recovering
5	Varol et al. [7]	14	M	Ataxia, hallucinations	135	3 days	10days (disappeared)	MIS-C	Recovering
6	Bektaş et al. [8]	11	F	Personality changes	132	6 days	7 days (disappeared)	MIS-C	Recovering
7	Bektaş et al. [8]	10	M	Agitation, disorient, hallucinations	131	2 days	7 days (disappeared)	MIS-C	Recovering
8	Gaur et al. [9]	12	M	Lethargy, headache, vomiting	NR	5 days	Not performed	MIS-C	Recovering
9	Gaur et al. [9]	9	M	Altered mental state, lethargy, dysarthric, ataxic	NR	NR	NR (disappeared)	COVID-19	Recovering
10	PR	9	M	Vomiting, poor speech	133	2 days	9 days (disappeared)	COVID-19	Recovering

All of these patients were older than nine years of age. Therefore, even in older children, MERS should be considered when SARS-CoV-2 infection is present. In the present case, encephalopathy was suspected because of poor speech. MRI performed on the second day of admission led to a diagnosis of MERS. In addition, follow-up MRI performed one week after admission confirmed the disappearance of high-signal areas in the corpus callosum, confirming the efficacy of the treatment. Thus, MRI is useful for diagnosing encephalopathy and determining the effectiveness of treatment. DWI is a key imaging modality used to diagnose MERS. It reveals homogeneously reduced diffusion in the corpus callosum without contrast enhancement [5]. Therefore, MRI should be considered when encephalopathy is suspected.

Transient signal changes in the splenium of the corpus callosum on MRI have been reported in various neurological and non-neurological disorders. Based on signal changes, the splenium is involved in two types according to its shape and extent: oval, circumscribed, with well‑defined borders usually located in the middle, or broader, with less regular borders and involving the entire splenium ("boomerang sign") [12].

MERS is caused by intramyelinic axonal edema or local inflammatory cell infiltration [3]. Takanashi et al. [13] have reported that most patients with MERS have mild hyponatremia. Hypotonic hyponatremia causes fluid accumulation in the brain, leading to cerebral edema, headache, nausea, vomiting, confusion, and seizures. It is clinically challenging to distinguish MERS from hyponatremic encephalopathy or to exclude hyponatremia as a causative factor of MERS [13]. Our previous reports revealed that MERS is not specifically associated with hyponatremia [14-16]. Previous SARS-CoV-2-associated MERS reports, summarized in Table 2, have also reported low to normal serum sodium levels. These findings indicate that SARS-CoV-2-associated MERS can develop without hyponatremia.

In adults, SARS-CoV-2 mainly causes respiratory infections but may also be accompanied by neurological symptoms and complications such as cerebral infarction, encephalitis, and encephalopathy have also been reported [17]. In recent years, acute encephalopathy caused by SARS-CoV-2 infection in Japanese children appeared as a primary neurological symptom before respiratory symptoms [18]. No respiratory symptoms were observed in this case, and obvious respiratory symptoms have accompanied no previous MERS cases. Thus, the mechanism of development of central nervous symptoms associated with SARS-CoV-2 infection is speculated to differ between adults and children.

In this case, neurological symptoms and MRI findings improved with the administration of mannitol and edaravone alone. However, except for the present case and patient 9 in Table 2, the other patients developed pediatric multisystem inflammatory syndrome and required methylprednisolone pulse therapy and intravenous immunoglobulin therapy. Furthermore, as for patient 2, the clinical symptoms can persist even in patients with MERS. Thus, although MERS is generally considered to have a good prognosis, careful follow-up is required.

## Conclusions

Performing an MRI evaluation is useful in children with SARS-CoV-2 infection in conjunction with impaired consciousness, poor speech, and behavior, given the possibility that they may be in the early stages of encephalopathy. DWI is an important imaging modality for diagnosing MERS, and performing MRI is recommended to detect reversible lesions in the corpus callosum. MRI is not only useful for diagnosing encephalopathy but also for assessing the effectiveness of treatment.
